# Danggui Buxue Tang Ameliorates Bleomycin-Induced Pulmonary Fibrosis by Suppressing the TLR4/NLRP3 Signaling Pathway in Rats

**DOI:** 10.1155/2021/8030143

**Published:** 2021-07-23

**Authors:** Jiepeng Wang, Hao Wang, Fang Fang, Chaoyi Fang, Shaoxian Wang, Chenxi Lu, Na Liu

**Affiliations:** ^1^School of Preclinical Medicine, Hebei University of Chinese Medicine, Shijiazhuang 050200, China; ^2^Hebei Key Laboratory of Integrated Chinese and Western Medicine for Lung Disease Research, Shijiazhuang 050091, China

## Abstract

**Objective:**

To investigate the effects of Danggui Buxue Tang (DBT) on rats with pulmonary fibrosis (PF) and the underlying mechanism.

**Methods:**

Sixty specific pathogen-free (SPF) male Sprague-Dawley (SD) rats were randomly divided into 4 groups: control, PF, prednisone treatment, and DBT treatment. Intratracheal instillation of bleomycin (BLM) was performed to establish a PF rat model. DBT was administered to PF rats concurrently for 2 weeks. Lung samples were then collected for HE and Masson staining after pulmonary function testing, and semiquantitative analysis for the degree of alveolitis and fibrosis was performed using the Szapiel and Ashcroft score systems. Myeloperoxidase (MPO) activity, hydroxyproline (HYP), hyaluronic acid (HA), and inflammatory cytokine content were measured. Western blotting was performed to detect fibrotic marker and TLR4/NLRP3 signaling pathway changes.

**Results:**

Oral administration of DBT attenuated weight loss, survival rate, and pulmonary index. Lung histopathologic lesions were also reduced. DBT inhibited PF by decreasing the secretion of inflammatory cytokines and collagen deposition. Specifically, DBT reduced tumor necrosis factor-alpha (TNF-*α*), interleukin 1 beta (IL-1*β*), IL-6, HYP, alpha-smooth muscle actin (*α*-SMA), collagen I, and collagen III levels. Corollary experiments identified a potential mechanism involving suppression of TLR4/MyD88/NF-*κ*B signaling pathway activation and the NLRP3/ASC/caspase-1 axis, the downstream regulatory pathway.

**Conclusion:**

DBT exhibited a potent effect on BLM-induced PF rats by inhibiting the TLR4/NLRP3 signaling pathway. Thus, DBT alleviates pulmonary inflammation to inhibit fibrotic pathology and should be considered as a candidate for the clinical treatment of PF.

## 1. Introduction

Pulmonary fibrosis (PF) is a fatal and incurable lung disease that is characterized by deposition of extracellular matrix (ECM) [1, 2]. The median survival time of PF is 2–5 years, and the main cause of death is impaired pulmonary function and respiratory failure induced by ECM with irreversible scarring [3, 4]. Few medications and limited therapeutics are available, although nintedanib and pirfenidone are administered but associated with side effects, such as liver damage and untoward gastrointestinal effects [5, 6].

The pathogenesis underlying PF has not been established. Smoking, environmental agents, and infections are thought to induce and contribute to the pulmonary inflammation leading to PF [7]. Recent studies have confirmed that the excessive release of inflammatory cytokines, such as tumor necrosis factor-alpha (TNF-*α*), interleukin 1 beta (IL-1*β*), and IL-6, are involved in the pathogenesis of PF [8]. Toll-like receptor 4 (TLR4) is an important mediator of inflammation and has been reported to activate both proinflammatory and profibrotic pathways in PF [9, 10]. In TLR4 knockout mice, PF has been shown to be significantly attenuated by inhibiting fibroblast activation and collagen production, while the upregulation of fibroblast TLR4 augments transforming growth factor-beta 1 (TGF-*β*1) sensitivity in bleomycin- (BLM-) induced PF [11]. Downstream cytokines of TLR4, such as myeloid differentiation primary response 88 (MyD88), nuclear factor kappa-B (NF-*κ*B), and inflammatory cytokines, are significantly activated in a PF murine model [12]. As the inflammatory cytokines mature, nucleotide-binding oligomerization domain- (Nod-) like receptor 3 (NLRP3) inflammasome activation is needed in PF [13, 14]. Additionally, NLRP3 regulates the epithelial-to-mesenchymal transition (EMT) via TGF-*β*1 [15], an important fibrogenic factor that stimulates differentiation of fibroblasts-to-myofibroblasts expressing alpha-smooth muscle actin (*α*-SMA) through the Smads signaling pathway and facilitates the accumulation of ECM [12].

Danggui Buxue Tang (DBT) is a traditional Chinese medicine recorded in the book of “Nei Wai Shang Bian Huo Lun” and described by Li Dongyuan in AD 1247 as a 5 : 1 mix of Radix Astragali and Radix Angelica Sinensis. Based on the critical role of inflammation during PF, inflammation may serve as a potential target for restraining the pathologic process. Clinical studies have confirmed the effects of DBT in inhibiting inflammatory cytokines, such as TNF-*α* and TGF-*β*1, in PF patients [16]. Pharmacologic studies have shown that DBT has antifibrosis effects in animal models. In our previous study, we have demonstrated that DBT could ameliorate BLM-induced PF rats by inhibiting pulmonary inflammation and collagen deposition [17], oxidative damage [18], and angiogenesis [19]. Additionally, astragaloside IV, one of the main active components of Astragali Radix, causes anti-inflammatory effects in a BLM-induced murine model [20] and Angelica polysaccharides have been identified to improve pulmonary function, lung indices, and bodyweight [21]. However, whether the specific mechanism by which DBT inhibits inflammation in animals with PF is related to TLR4/NLRP3 signaling pathway, which mediates inflammation in PF, remains unknown and thus warrants further research.

The present study aimed to determine the mechanism by which DBT ameliorates BLM-induced PF in rats by examining TLR4/NLRP3 signaling pathway changes and determining the anti-inflammatory and antifibrotic effects.

## 2. Materials and Methods

### 2.1. Animals

Sixty specific pathogen-free (SPF) male Sprague-Dawley (SD) rats, weighing 180–200 g, were obtained from Beijing Vital River Laboratory Animal Technology Co., Ltd. (certificate number SCXK2016-0006, Beijing, China). All rats were housed at 22–26°C with a relative humidity of 45%–55% and a 12 h light/dark cycle. The rats were provided a pellet diet and tap water *ad libitum*. In this study the rats were cared for according to the *Guide for the Care and Use of Laboratory Animals* (The National Academies Press, Washington, DC: revised 1996, publication no. 85–23). All experiments were approved by the Ethics Committee for Animal Experiments of the Hebei University of Chinese Medicine (No. DWLL2018024).

### 2.2. Preparation of DBT

DBT herbs were purchased from Guangdong Yifang Pharmaceutical Co., Ltd. (Foshan, China; Table 1). The two Chinese Materia Medica, Huangqi (Radix Astragali) and Danggui (Radix Angelica Sinensis), were dissolved with deionized water at a 5 : 1 ratio. The basic pharmacodynamic material of DBT consists of saponin (astragaloside I, II, IV, V, VI, and VII; isoastragaloside II; and acetylastragaloside I), flavonoids (formononetin, ononin, calycosin, and calycosin-7-O-*β*-D glucoside), volatile oils (Z-ligustilide, E-ligustilide, 3-butylidenephthalide, n-butylphthalide, and *α*-pinene), organic acids (ferulic acid, chlorogenic acid, isochlorogenic acid, vanillic acid, and azelaic acid), and polysaccharides (astragalus and Angelica polysaccharides) [22].

### 2.3. Animal Modeling and Drug Administration

A rat model of PF was induced by intratracheal instillation of BLM (Nippon Kayaku, Tokyo, Japan), as described previously [23, 24]. In brief, after a 1-week acclimatization, the rats were randomly divided into 4 groups according to the bodyweight: sham (*n* = 15), model (*n* = 15), positive drug (*n* = 15), and DBT (*n* = 15). The rats of model, positive drug, and DBT were anesthetized with 2% pentobarbital sodium, then BLM (5 mg/kg) was administered intratracheally, while the rats of control were given normal saline solution intratracheally. Two days after surgery, DBT (0.81 g/kg/d (calculated based on a 60 kg bodyweight for adults) was administered orally to rats once daily for 14 consecutive days. Prednisone (0.5 mg/100 g, Zhejiang Xianju Pharmaceutical Co., Ltd., Xianju, China) was used as a positive drug. The model and sham group rats were given the same volume of 0.9% normal saline solution. The bodyweight and food intake were recorded daily. Mortality was documented to calculate the survival rate.

### 2.4. Pulmonary Function Tests

After the last administration of DBT, pulmonary function tests were performed as described previously [25]. Spontaneous breathing was suppressed by the intraperitoneal injection of pentobarbital (60 mg/kg). As the animals were unconscious, the trachea was exposed and a 0.2 cm transverse incision was made. Then, a special tracheal needle was placed into the trachea and a sterile surgical suture was used to fix the needle. The rats were then connected to the flexiVent system (fx4, SCIREQ, Montreal, Canada). The system was calibrated according to instrument manuals before detection. A total lung capacity (TLC) perturbation was performed to normalize the lungs before collecting the data. The snapshot and primewave (Prime-8) perturbation were performed until 3 acceptable measurements (coefficient of determination > 0.95) were recorded in each rat, from which an average was calculated. In snapshot perturbation, total respiratory resistance (Rrs), elastance (Ers), and compliance (Crs) were documented, while in Prime-8, perturbation Newtonian airway resistance (Rn), tissue damping (G), and tissue elastance (H) were calendared, according to the literature [26].

### 2.5. Tissue Collection

After lung function was determined, the animals were sacrificed by decapitation while unconscious. Ultimately, the lungs were removed *en bloc*, washed in cold isotonic saline, and then immediately weighed to compute the pulmonary index (pulmonary wet weight (mg)/bodyweight (g) × 100%). Subsequently, three rats in each group were randomly selected and bronchoalveolar lavage (BAL) was performed by cannulating the trachea with a 12^#^ mouse gavage needle to collect bronchoalveolar lavage fluid (BALF) from the left lung, and then the tissue was extirpated. The left lung tissues from another 5 rats were fixed in 4% phosphate-buffered paraformaldehyde for histopathologic examination. The entire right lung and the remaining left lung were immediately frozen with liquid nitrogen and stored at −80°C for further analysis.

### 2.6. Histopathologic Examination

The lung was fixed in paraformaldehyde for 24 h before routine dehydration and paraffin embedding. Then, the tissue was sliced in 4 µm thick sections for H&E and Masson-trichrome staining to evaluate inflammation and pathologic changes, as well as collagen deposition, respectively. To semiquantify the histopathologic changes, the Szapiel score was used to quantify alveolitis, and the Ashcroft score was used to quantify pulmonary lesions [27, 28]. The scoring standards are shown in Tables 2 and 3 . The collagen volume fraction (CVF) was calculated using Image *J* software (version 1.52 h; https://imagej.nih.gov/ij, NIH, Bethesda, MD, USA) to assess the degree of lung devastation [CVF (%) = collagen area/tissue area] [29]. All procedures were performed at 200 × magnification through an optical microscope (Olympus, Tokyo, Japan); 3 areas were randomly selected in 1 section, and 6 sections were randomly selected for analysis. The scores were assessed separately by two experimentalists.

### 2.7. Differential Cell Count in BALF

To obtain BALF, 1 mL of 0.9% normal saline was instilled into the lungs 5 times through a tracheal cannula, and an average of 4 mL of bronchial lavage was collected. Then, the samples were centrifuged immediately at 3000 rpm for 5 min at 4°C. The supernatant fluid was removed and approximately 50 *μ*L of each sample was reserved to smear on the slide in an area of approximately 1 cm^2^ with 2 pieces in 1 slice. Subsequently, the smear was stained with Wright-Giemsa stain (Baso Diagnostics, Inc., Zhuhai, China) and the total cells and cell lines (macrophages, lymphocytes, and neutrophils) were enumerated and identified in a single-blind fashion under oil lens by Professor Huazhou Xu and experimentalist Fang Fang, who are technicians at the Experimental Center of Hebei University of Chinese Medicine. A total of 200 leukocytes were counted.

### 2.8. Content of Hydroxyproline (HYP) and Hyaluronic Acid (HA)

To measure the HYP (Nanjing Jiancheng Bioengineering Institute, Nanjing, China) and HA levels (Beijing North Biotechnology Research Institute Co., Ltd., Beijing, China), 100 mg of lung tissue was weighed and homogenized. Sample alkali hydrolysis was performed to determine the content of HYP, 1 mL of hydrolytic liquid was added, and the mixture was boiled in water for 20 min in accordance with the manufacturer's instructions. The absorbance value at 550 nm was determined. Radioimmunoassay (RIA) was performed to measure the HA level.

### 2.9. Myeloperoxidase (MPO) Activity Examination

Wet lung tissue (100 mg) was homogenized with reagent provided with the MPO examination kit (Nanjing Jiancheng Bioengineering Institute) at a 1 : 19 ratio before boiling in water at 37°C for 15 min. Then, reagents were added and fixed according to the instruction manual. Ultimately, the samples were heated for 10 min in a 60° water bath, and absorbance was measured at 460 nm.

### 2.10. Enzyme-Linked Immunosorbent Assay (ELISA) for Measuring Inflammatory Cytokines

ELISA was performed to measure the content of TNF-*α*, IL-6, and IL-1*β* in lung tissues. Wet lung tissue (100 mg) was homogenized, centrifuged at 4°C (12,000 rpm for 15 min), and the supernatant was collected. The inflammatory cytokine content was examined according to the manufacturer's protocol. The ELISA kits were purchased from IBL International GmbH (Hamburg, Germany). The optical density was determined at 450 nm on a spectrophotometer (Molecular Devices Corporation, Sunnyvale, CA, USA).

### 2.11. Western Blotting Analysis

Lung tissues were homogenized in 200 *μ*l of RIPA lysis buffer (Thermo Fisher Scientific, Waltham, MA, USA) containing 0.1% PMSF (Beijing Solarbio Science and Technology Co., Ltd., Beijing, China). A BCA protein detection kit (Beijing Solarbio Science and Technology Co., Ltd.) was used to determine the protein concentration. Proteins (30 *μ*g) were separated by sodium dodecyl sulfate-polyacrylamide gel electrophoresis (SDS-PAGE) in running buffer and transferred onto a polyvinylidene difluoride (PVDF) membrane (Merck Millipore, Darmstadt, Germany) using a semidry transfer (Bio-Rad, Los Angeles, California, USA). Nonspecific binding was blocked using 5% skim milk powder (Biofroxx, Germany) in TBST, after which the membranes were incubated with primary antibodies overnight at 4°C. The antibodies were against collagen I (1 : 1000; Abcam, Cambridge, UK, USA), collagen III (1 : 1000; Abcam), *α*-SMA (1 : 500; Abcam), TLR4 (1 : 1000; Thermo), MyD88 (1 : 500; Thermo), NF-*κ*B (1 : 500; Thermo), *p*-NF-*κ*B (1 : 500; Abcam), NLRP3 (1 : 1000; Abcam), cleaved caspase 1 (1 : 500; Cell Signaling), ASC (1 : 1000; Thermo), and GAPDH (1 : 5000; Abcam). After washing thrice with TBST, the membranes were incubated with horseradish peroxidase- (HRP-) conjugated goat anti-rabbit/mouse secondary antibody (1 : 5000 dilution in 5% skimmed milk powder in TBST, Bioeasy, Shenzhen, China) at room temperature for 1 h. The immunoreactive proteins were visualized using an enhanced chemiluminescence reagent (Vazyme, Nanjing, China) and captured as a digital image. The images were developed using the Fusion FX5 Spectra Imaging System (Vilber Loumat, Paris, France). The gray value of the protein bands was detected using Image-Pro Plus 6.0 software, and the target band-to-GAPDH ratio was used for semiquantitative analysis.

### 2.12. Statistical Analysis

Firstly, the normal distribution and homogeneity tests were performed. If the data were normally distributed and homogenous, one-way analysis of variance (ANOVA) was performed, followed by the post hoc least significant difference (LSD) test for pairwise comparisons. The data are expressed as a mean ± standard deviation (SD). If the data were not normally distributed and homogenous, the Kruskal–Wallis H test was used and the Nemenyi method was adopted for a comparison between groups. The data are shown as a median and interquartile range. The repeated measurements data were analyzed by repeated measures ANOVA. Fisher's exact test was used for mortality analysis. All statistical analyses were performed using the SPSS software package (IBM SPSS Statistics for Windows, version 21.0, Armonk, NY, USA).

## 3. Results

### 3.1. Effect of DBT on Bodyweight, Food Intake, Pulmonary Index, and Survival Time

**Figure 1:**
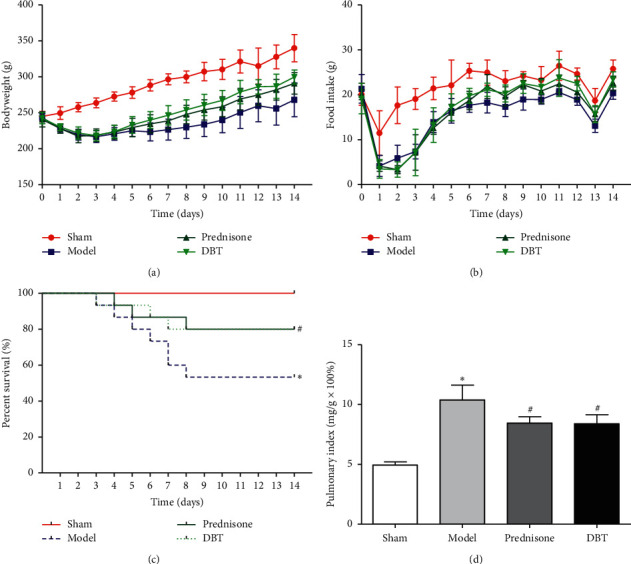
Effect of DBT on bodyweight, food intake, survival time, and pulmonary index in PF rats. (a) Bodyweight changes in 4 groups (*n* = 8–15). (b) Food intake changes in 4 groups (*n* = 6). (c) Percent survival in 4 groups (*n* = 15). (d) Pulmonary index changes in 4 groups (*n* = 8). ^*∗*^*P* < 0.05 versus the sham group and ^#^*P* < 0.05 versus the model group.

As shown in Figure 1 and Tables 4 and 5, there was a significantly reduced bodyweight, food intake, and percent survival after intratracheal instillation by BLM with a dosage of 5 mg/kg compared with the sham group. In contrast, the pulmonary index of the model group was increased. After being treated with DBT (0.81 g/kg/d), the weight and food intake were higher than the model rats, and the percent survival was improved. In addition, DBT also reduced the pulmonary index.

### 3.2. Effect of DBT on Pulmonary Function

**Figure 2:**
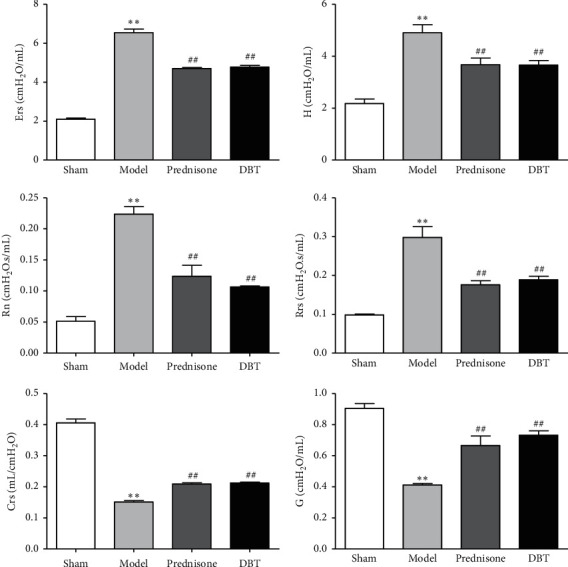
Effect of DBT on pulmonary function in rats with PF. Ers: elastance; H: tissue elastance; Rn : Newtonian airway resistance; Rrs: total respiratory resistance; Crs: compliance; G: tissue damping; *n* = 6 for each group. ^*∗∗*^*P* < 0.01 versus sham group and ^##^*P* < 0.01 versus model group.

To determine the effect of DBT on respiration in rats with PF, a pulmonary function test was conducted using a flexiVent system. As shown in Figure 2, Ers, H, Rn, and Rrs in the model rats were markedly increased compared with the sham operation rats, while airway compliance (Crs and G) was decreased. In contrast, the DBT-treated groups presented a significant decrease in airway resistance (Ers, H, Rn, and Rrs) and an increase in airway compliance (Crs and G) compared to the BLM control group.

### 3.3. Effect of DBT on Pulmonary Inflammation in Rats with PF

**Figure 3:**
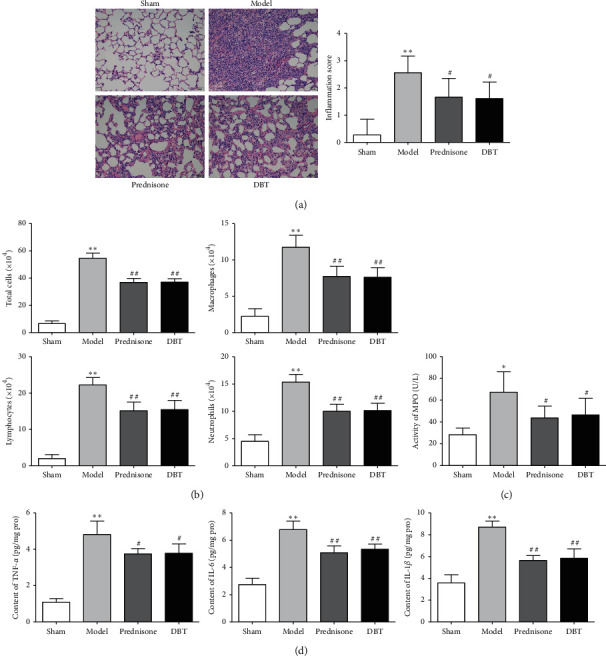
Effect of DBT on pulmonary inflammation in rats with PF. (a) Pathologic changes of lung tissues evaluated by H&E staining (scale bar = 50 *μ*m) and assessed by the Szapiel score (*n* = 18). (b) Cell counts and classification in BALF (*n* = 8). (c) MPO activity tested by an examination kit (*n* = 8). (d) Levels of TNF-*α*, IL-6, and IL-1*β* in lung tissues examined by ELISA (*n* = 8). ^*∗*^*P* < 0.05 and ^*∗∗*^*P* < 0.01 versus sham group; ^#^*P* < 0.05 and ^##^*P* < 0.01 versus model group.

As BLM-induced PF often progresses with inflammatory responses, we next determined if the antifibrotic efficacy of DBT was attributed to anti-inflammation activity. Pathologic changes in lung tissues were evaluated by H&E staining. As shown in Figure 3(a), the sham group showed complete lung tissue structure and no thickening of the alveolar septum. The BLM control group, in contrast, had severe damage to the alveolar structure, and the alveolar septum was significantly widened with a large inflammatory cell infiltration and fibroblast proliferation compared with the control group. Additionally, the inflammation score was significantly higher than the sham group, while the DBT treatment group was improved.

BALF was collected and counted to investigate the effect of DBT on inflammatory cell infiltration. As shown in Figure 3(b), BLM treatment dramatically increased the number of total cells, macrophages, lymphocytes, and neutrophils in comparison with the sham group. DBT attenuated the aforementioned phenomena. In the DBT group, the number of total cells, macrophages, lymphocytes, and neutrophils in BALF were reduced.

To further explore the mechanism by which DBT inhibits pulmonary inflammation in rats with PF, MPO activity and the levels of inflammatory factors (TNF-*α*, IL-6, and IL-1*β*) were determined. As shown in Figures 3(c) and 3(d), when compared with the sham group, MPO activity and the levels of TNF-*α*, IL-6, and IL-1*β* were increased; however, treating the PF rats with DBT (0.81 g/kg/d) for 2 weeks significantly suppressed MPO activity and the levels of inflammatory mediators (TNF-*α*, IL-6, and IL-1*β*).

### 3.4. Effect of DBT on Fibrotic Markers and Collagen Production in BLM-Induced Pulmonary Fibrosis

**Figure 4:**
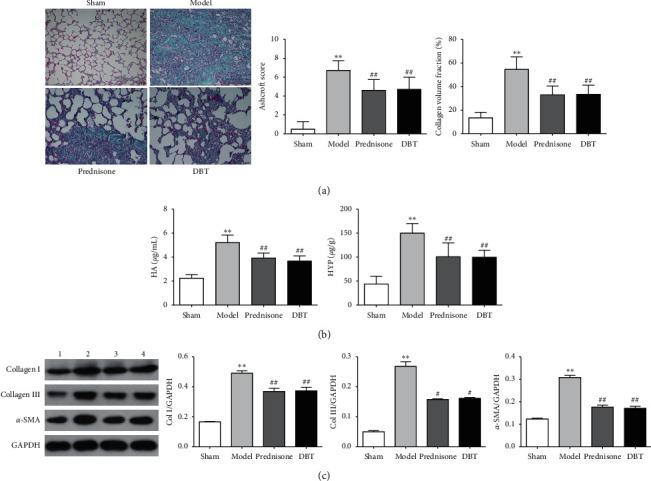
Effect of DBT on fibrotic markers and collagen production in rats with PF. (a) Masson-trichrome staining of lung tissues (scale bar = 50 *μ*m) and Ashcroft scores (*n* = 18). (b) HA and HYP levels in lung tissue (*n* = 8). (c) Alpha-SMA, collagen I, and collagen III proteins examined by Western blotting (*n* = 3). 1, sham group; 2, model group; 3, prednisone group; 4, DBT group. ^*∗∗*^*P* < 0.01 versus sham group; ^#^*P* < 0.05 and ^##^*P* < 0.01 versus model group.

To further determine whether DBT inhibits collagen production, Masson-trichrome staining was applied. As shown in Figure 4(a), in BLM-stimulated rats, the lung tissue exhibited a large amount of blue collagen deposition and marked increase in the area of collagen fibers and fibrosis scores, indicating that BLM-induced severe lung fibrosis; however, the sham group was intact with a thin collagenous layer of the bronchus wall in the lung tissue. DBT had a protective effect. After treatment with DBT, the collagen fiber area and fibrosis scores were lower than that of the rats with fibrosis treated with normal saline.

The HYP and HA levels were widely examined to determine collagen levels in tissue samples with fibrosis. As shown in Figure 4(b), the high levels of HYP and HA in rats with BLM-induced PF were clearly reversed by DBT administration.

Alpha-SMA represents the primary effector cells that generate the ECM and provide contractile forces during fibrogenesis, while collagens I and III are interstitial collagens and contribute to ECM deposition. In the current study, Western blotting was used to determine the expression of *α*-SMA, collagen I, and collagen III. As shown in Figure 4(c), upon BLM treatment, *α*-SMA, collagen I, and collagen III protein levels were augmented. Nevertheless, DBT profoundly suppressed the expression of the ECM.

### 3.5. Effect of DBT on the TLR4/MyD88/NF-*κ*B Signaling Pathway

**Figure 5:**
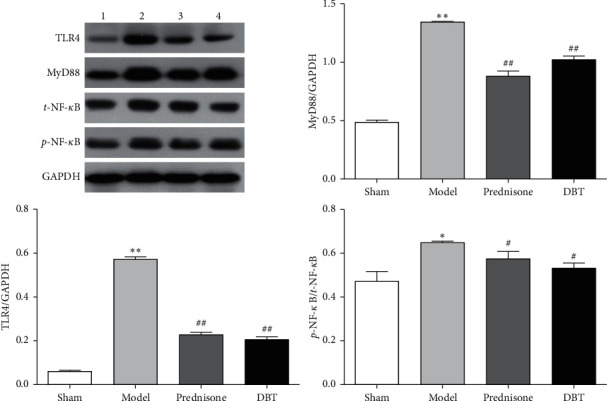
Effect of DBT on the TLR4/MyD88/NF-*κ*B signaling pathway. Western blotting was used for analysis. 1, sham group; 2, model group; 3, prednisone group; and 4, DBT group. ^*∗*^*P* < 0.05 and ^*∗∗*^*P* < 0.01 versus sham group; ^#^*P* < 0.05 and ^##^*P* < 0.01 versus model group; *n* = 3 in each group.

It is well-known that activation of the TLR4/MyD88/NF-*κ*B signal pathway promotes and amplifies the inflammatory response during inflammation. To monitor whether the TLR4/MyD88/NF-*κ*B signaling pathway was involved in the fibrotic process and explore the role of DBT in its modulation, Western blotting was used to determine the expression of TLR4, MyD88, *t*-NF-*κ*B, and phosphorylation of NF-*κ*B (*p*-NF-*κ*B). As shown in Figure 5, we detected higher expression of TLR4, MyD88, and the ratio of *p*-NF-*κ*B to *t*-NF-*κ*B in the BLM control group; however, after treatment with DBT, the expression of TLR4, MyD88, and the ratio of *p*-NF-*κ*B to *t*-NF-*κ*B were gradually decreased.

### 3.6. Effect of DBT on the NLRP3/ASC/Caspase 1 Signaling Pathway

**Figure 6:**
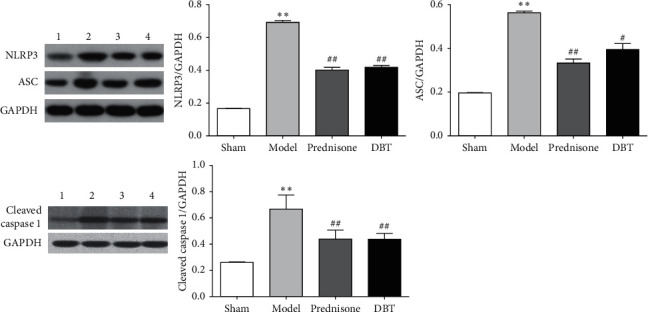
Effect of DBT on the NLRP3/ASC/caspase 1 signaling pathway. Western blotting was used for analysis. 1, sham group; 2, model group; 3, prednisone group; and 4, DBT group. ^*∗∗*^*P* < 0.01 versus sham group; ^#^*P* < 0.05 and ^##^*P* < 0.01 versus model group; *n* = 3 in each group.

Studies have indicated that the NLRP3 inflammasome acts as an important signaling molecule downstream of TLR4 and participates in the progress of PF. We thus determined the NLRP3 inflammasome level in the lung tissue to clarify whether the anti-inflammatory effects of DBT are related to the NLRP3 inflammasome. As shown in Figure 6, BLM exposure contributed to a significant increase in the levels of NLRP3, ASC, and cleaved caspase 1 protein. Interestingly, DBT treatment suppressed this cascade in rats with PF.

## 4. Discussion

PF is a progressive and devastating pulmonary parenchymal disease with a poor prognosis and no curative therapies. PF is characterized by excessive matrix deposition that disrupts the normal architecture of the lung parenchyma [30]. Therefore, preclinical experimental studies are urgently needed. The BLM-induced PF model is the most commonly used *in vivo* system for investigating candidate therapies because the toxicity predominantly affects the lungs [30, 31]. During the pathologic progression of the animal model, it was found that gradual subsidence of the inflammatory response with an accompanying increase in fibroproliferation 7–14 days post-BLM [31]. This finding indicates that during this period lung tissue presents inflammation accompanied by interstitial collagen deposition. Thus, this can be used to predict whether corresponding treatment therapies are likely to inhibit inflammation relating to fibrosis. The present study showed that, compared with controls, rats with PF have a higher mortality and lung index, lower bodyweight gain, food intake, and pulmonary dysfunction. Additionally, H&E and Masson-trichrome staining demonstrate cell infiltration and collagen deposition in the lungs of the model rats. These findings are consistent with previous studies [12, 25, 26, 32], which suggests that the PF rat model was successfully established in this study.

Tissue damage and inflammation are important triggers for fibrosis [33]. Clinical and experimental studies have shown inflammatory factors released and assembled in PF, such as TNF-*α*, IL-6, and IL-1*β* [34, 35]. In rats with PF, BLM is reverted to a free radical, which reacts with oxygen to form superoxide anion, and then superoxide anion can produce more active oxygen (e.g., hydrogen peroxide and hydroxyl radical) to damage alveolar epithelial and vascular endothelial cell, thus disrupting the oxidation/antioxidant system balance to generate inflammatory cells [36]. MPO is an enzyme that scavenges reactive oxygen to prevent excessive accumulation of reactive oxygen [37]. In PF models and patients, MPO activity was enhanced, indicating antioxidant enzyme activity and the ability to scavenge oxygen-free radicals was inhibited, resulting in the accumulation of excess oxygen free radicals to cause lung damage [38, 39]. Inflammatory cells produce chemokines and cytokines (e.g., TNF-*α*, IL-1*β*, and IL-6) to participate during repairing and reconstructing the lung in the pathologic process of PF [40, 41]. TGF-*β*1 is the backbone of fibroblast repair responses and is a key profibrotic factor secreted by macrophage cells that can regulate the migration, proliferation, and differentiation of fibroblasts [42]. Fibroblasts can transdifferentiate into myofibroblasts upon activation, which is characterized by overexpression of *α*-SMA and is considered as a marker of myofibroblast and deposition of ECM [43] and primarily collagen types I and III. The TGF-*β* mediated Smads signaling pathway is a pivotal mechanism in the development of PF [44]. When TGF-*β*1 is stimulated, TGF-*β*1 binds to the type II TGF-*β* receptor and triggers the autophosphorylation of the type I TGF-*β* receptor to active Smad2/3, promoting fibroblast proliferation, differentiation, and ECM remodeling [45, 46]. In this study, the cell count of total cells, macrophages, neutrophils, and lymphocytes were increased among the rats with PF. In addition, the levels of TNF-*α*, IL-1*β*, and IL-6 proteins detected by ELISA were increased compared with the sham group, and MPO exhibited the same trend. Moreover, *α*-SMA expression was augmented. Furthermore, the content of HYP, a unique component of collagen fibers and an important indicator to measure the metabolism of collagen tissues [47], was increased relative to the control group. These results demonstrate that rats with PF have inflammatory infiltration and accumulation of excess oxygen-free radicals, which contributes to the tissue destruction and structural remodeling of the lung. This is followed by collagen deposition, which is characterized by the increased content of HYP and expression of *α*-SMA, collagen I, and collagen III, which can be attributed to activation of the TGF-*β*/Smad3 pathway. Thus, aiming to repress the release and secretion of inflammatory factors might be an efficacious approach to managing PF.

Previous studies have suggested that the immune system is involved in lung fibrosis [48]. TLRs represent a conserved family of innate immune recognition receptors that can regulate innate and adaptive immune responses [49] and are involved in noninfectious inflammatory diseases [50]. Recent studies have suggested that TLR4 activity is critical for inflammation and PF, in both basic and clinical research [51, 52]. The pattern recognition receptor, TLR4, can be activated by many endogenous damage-associated molecular patterns (DAMPs) present as a result of cellular damage, such as HA synthesis by fibroblasts [53]. After binding to TLR4, two critical intracellular signaling pathways are triggered, including the MyD88-dependent and MyD88-independent signaling cascades [54, 55]. The MyD88-dependent signal transduction activates NF-*κ*B through activation of its inhibitory protein (nuclear factor-kappa B inhibitor protein [I*κ*B*α*]), which allows NF-*κ*B nuclear translocation and controls expression of proinflammatory cytokines, such as TNF-*α*, IL-1*β*, and IL-6 [56].

In addition to the TLR4/MyD88 signaling pathway, a large multimeric protein complex, known as an inflammasome, also governs the production of proinflammatory cytokines [57]. This complex has three components: NLR, adaptor molecule-apoptosis associated speck-like protein (ASC), and caspase 1. Among the NLR family, NLRP3 has been reported to participate in PF [15]. For example, after silencing NLRP3, E-cadherin expression is increased, and *α*-SMA and TGF-*β*1 are reduced, indicating that NLRP3 regulates the EMT [15]. Moreover, NLRP3 and caspase 1 levels have been reported to be increased in the untreated macrophages in BALF of patients with PF, as are the IL-1*β* levels [58]. Additionally, the NLRP3 inflammasome is an important signaling molecule downstream of TLR4, which can mediate NF-*κ*B signaling to promote NLRP3 inflammasome priming and procaspase 1 clipped into cleaved caspase 1, an activated form of procaspase 1, which leads to the maturation of IL-1*β* [59, 60].

The current study showed that the content of HA is increased in rats of PF. In addition, we observed that the TLR4/MyD88 and NLRP3/ASC/caspase 1 signaling cascade of model rats were activated. The results indicated that the TLR4/NLRP3 signaling pathway mediates inflammatory factor release and ECM deposition. Therefore, TLR4/NLRP3 is a potential target for treating PF.

DBT is a simple Chinese herbal formula that is widely used by women in China to relieve menopausal symptoms [61]. Studies have shown that DBT has benign effects on antifibrosis [62], and there are no reported side effects to date [63]. Regarding the BLM-induced PF model, research has confirmed that DBT inhibits oxidative stress by suppressing protein kinase D1 (PKD1)/NF-*κ*B/manganese superoxide dismutase (MnSOD) signaling pathway [18], restraining the synthesis of ECM, and balancing the metalloproteinase (MMP)/tissue inhibitor of metalloproteinase 1 (TIMP-1) system [64]. Moreover, our previous research had confirmed that DBT could decrease the content of TNF-*α*, IL-1*β*, and IL-6 through inhibiting TGF-*β*1/Smad3/plasminogen activator inhibitor 1 (PAI-1) signaling pathway to suppress PF [17]. The results imply that DBT relieves PF by restraining inflammation stimulated by ECM deposition, but the specific mechanism has not been established. This study illustrated that DBT improves the bodyweight, food intake, pulmonary function, and pulmonary index and prolongs the survival time in rats with PF. Indeed, this herbal medicine can ameliorate pulmonary lesions as it downregulates alveolitis and the Ashcroft score, the underlying mechanism for this might relate to the downregulation of MPO, *α*-SMA, HYP, collagen I, collagen III, and inflammatory factors (TNF-*α*, IL-1*β*, and IL-6). In short, DBT inhibits the pathologic progression of PF by suppressing inflammatory factor secretion and collagen deposition. To delineate the underlying mechanism, we next probed whether DBT has an ameliorating effect on the TLR4/NLRP3 signaling pathway. We found that after DBT treatment, the expression of TLR4, MyD88, the ratio of *p*-NF-*κ*B to *t*-NF-*κ*B, NLRP3, ASC, and cleaved caspase 1 were decreased. These results suggest that DBT inhibits PF by suppressing the TLR4/NLRP3 signaling transduction pathway.

There were also some limitations in this study. The concentration gradient of DBT was not performed in this study. The effect of DBT alone without BLM should also be examined in corollary *in vivo* studies.

## 5. Conclusions

Collectively, our findings demonstrated that DBT repressed BLM-induced PF *in vivo*. The mechanism might be due to inhibition of the TLR4/NLRP3 cascade. These findings suggest that DBT can be used as a potential therapeutic agent in the treatment of patients with idiopathic PF.

## Figures and Tables

**Table 1:** DBT ingredients.

Chinese name	Latin name	Voucher specimens	Packing size (g/package)	Equivalent to crude drug (g)
Huangqi	Radix Astragali	8017093	200	1000
Danggui	Radix Angelica Sinensis	8032653	200	660

**Table 2:** Szapiel score system.

Degree of alveolitis	Histopathologic features
0	No alveolitis
1	Thickening of the alveolar septum by a mononuclear cell infiltrate, with involvement limited to focal, pleural-based lesions occupying less than 20% of the lung and with good preservation of the alveolar architecture
2	A more widespread alveolitis involving 20%–50% of the lung, although still predominantly pleural based
3	A diffuse alveolitis involving >50% of the lung, with occasional consolidation of air spaces by the intra-alveolar mononuclear cells and some hemorrhagic areas within the interstitium and/or alveolus

**Table 3:** Ashcroft score system.

Grade of fibrosis	Histologic features
0	Normal lung
1	Minimal fibrous thickening of alveolar or bronchiolar walls
2	
3	Moderate thickening of walls without obvious damage to lung architecture
4	
5	Increased fibrosis with definite damage to lung structure and formation of fibrous bands or small fibrous masses
6	
7	Severe distortion of the structure and large fibrous areas; “honeycomb lung” is placed in this category
8	Total fibrous obliteration of the field

*Note.* If there was any difficulty in deciding between two odd-numbered categories, the field would be given the intervening even-numbered score.

**Table 4:** Comparison of bodyweight changes in each group (g, $\bar{x}$ ± *s*).

Day	Sham (*n* = 15)	Model (*n* = 8)	Prednisone (*n* = 12)	DBT (*n* = 12)
Day 0	245.07 ± 6.85	243.39 ± 5.55	240.68 ± 10.49	242.49 ± 7.73
Day 1	249.56 ± 8.82	228.04 ± 5.54^*∗∗*^	228.11 ± 6.57	230.17 ± 4.68
Day 2	257.87 ± 6.41	219.61 ± 7.47^*∗∗*^	217.37 ± 9.05	221.86 ± 8.39
Day 3	263.75 ± 6.77	216.69 ± 8.27^*∗∗*^	218.77 ± 8.70	218.26 ± 7.24
Day 4	272.65 ± 6.36	220.65 ± 8.81^*∗∗*^	223.06 ± 9.57	223.79 ± 8.62
Day 5	278.17 ± 7.87	225.03 ± 8.13^*∗∗*^	229.42 ± 12.91	233.19 ± 11.60
Day 6	288.3 ± 7.87	223.40 ± 12.59^*∗∗*^	234.58 ± 12.59^#^	239.9 ± 11.70^##^
Day 7	296.59 ± 7.66	226.65 ± 14.71^*∗∗*^	238.76 ± 10.77^#^	246.58 ± 13.47^##^
Day 8	299.95 ± 8.15	229.94 ± 15.56^*∗∗*^	247.68 ± 12.89^##^	253.99 ± 14.05^##^
Day 9	307.31 ± 12.86	233.94 ± 17.01^*∗∗*^	254.08 ± 14.13^##^	260.93 ± 14.86^##^
Day 10	310.39 ± 13.96	239.99 ± 17.75^*∗∗*^	258.44 ± 13.83^##^	267.21 ± 13.80^##^
Day 11	321.42 ± 15.91	250.36 ± 22.25^*∗∗*^	269.12 ± 15.09^#^	278.93 ± 16.37^##^
Day 12	315.13 ± 25.15	259.73 ± 22.21^*∗∗*^	274.92 ± 14.58	286.23 ± 9.95^##^
Day 13	327.83 ± 16.59	255.95 ± 23.04^*∗∗*^	281.99 ± 14.24^##^	286.93 ± 16.80^##^
Day 14	340.04 ± 18.99	267.99 ± 23.53^*∗∗*^	290.94 ± 14.91^##^	299.83 ± 9.75^##^

*Note.* ^*∗∗*^ *P* < 0.01 versus sham group; ^#^*P* < 0.05 and ^##^*P* < 0.01 versus model group.

**Table 5:** Comparison of food intake changes in each group (g, $\bar{x}$ ± *s*).

Day	Sham (*n* = 6)	Model (*n* = 6)	Prednisone (*n* = 6)	DBT (*n* = 6)
Day 0	20.11 ± 2.44	21.32 ± 3.18	20.00 ± 1.08	19.14 ± 3.39
Day 1	11.47 ± 4.95	4.16 ± 2.34^*∗∗*^	4.12 ± 1.17	3.50 ± 2.08
Day 2	17.65 ± 4.08	5.87 ± 2.91^*∗∗*^	3.34 ± 0.92	3.34 ± 1.71
Day 3	19.07 ± 2.31	7.35 ± 1.63^*∗∗*^	7.16 ± 3.95	7.17 ± 5.17
Day 4	21.43 ± 2.51	13.90 ± 2.33^*∗∗*^	12.58 ± 1.36	13.24 ± 3.86
Day 5	22.11 ± 5.63	16.42 ± 2.74^*∗*^	16.03 ± 1.86	17.23 ± 2.97
Day 6	25.34 ± 1.64	17.67 ± 1.59^*∗∗*^	18.73 ± 2.02	19.77 ± 1.64^#^
Day 7	24.94 ± 2.80	18.25 ± 2.29^*∗∗*^	21.85 ± 2.66^#^	21.12 ± 1.47^#^
Day 8	23.06 ± 2.31	17.30 ± 2.15^*∗∗*^	19.64 ± 2.12	20.27 ± 1.88^#^
Day 9	24.18 ± 0.95	18.95 ± 2.35^*∗∗*^	22.24 ± 0.87^##^	22.49 ± 2.18^##^
Day 10	23.26 ± 3.04	18.93 ± 1.01^*∗∗*^	20.82 ± 2.76	21.77 ± 1.58^#^
Day 11	26.45 ± 3.26	20.53 ± 0.83^*∗∗*^	22.42 ± 1.97	23.74 ± 4.06^##^
Day 12	24.63 ± 1.35	18.90 ± 1.23^*∗∗*^	20.57 ± 1.59	22.49 ± 1.59
Day 13	18.71 ± 2.69	13.09 ± 1.47^*∗*^	15.68 ± 1.63	15.68 ± 1.91
Day 14	25.77 ± 1.98	20.38 ± 1.39	22.59 ± 1.55^#^	23.42 ± 1.69^##^

*Note.* ^*∗*^ *P* < 0.05 and ^*∗∗*^*P* < 0.01 versus sham group; ^#^*P* < 0.05 and ^##^*P* < 0.01 versus model group.

## Data Availability

The data used to support the findings of this study are available from the corresponding author upon request.
